# Posturographic analysis of older adults without dementia and patients with Alzheimer’s disease: A cross-sectional study

**DOI:** 10.1590/1980-57642018dn13-020008

**Published:** 2019

**Authors:** Paula Sant’Anna, Felipe de Oliveira Silva, Ana Carolina de Mello Alves Rodrigues, Jéssica Plácido, José Vinicius Ferreira, Estele Caroline Welter Meereis, Jomilto Praxedes, Valeska Marinho, Jerson Laks, Renato Sobral, Andrea Camaz Deslandes

**Affiliations:** 1 Institute of Psychiatry, Universidade Federal do Rio de Janeiro, RJ, Brazil.; 2 Post-graduate Program of Health Sciences, Universidade Estadual de Montes Claros, MG, Brazil.; 3 Universidade Federal do Rio de Janeiro, RJ, Brazil.; 4 Universidade do Estado do Rio de Janeiro, RJ, Brazil.

**Keywords:** motor biomarker, Alzheimer’s disease, postural control, balance, dementia, older adults, biomarcador motor, doença de Alzheimer, controle postural, equilíbrio, demência, idosos

## Abstract

Additional clinical tools should be investigated to facilitate and aid the early diagnosis of cognitive decline. Postural control worsens with aging and this may be related to pathological cognitive impairment.

**Objective:**

to compare the balance of older adults without dementia in a control group (CG) and with Alzheimer’s disease (AD), to observe the possible association with the independent variables (diagnosis, age, gender, and global cognition) and to verify the best posturographic analyses to determine the difference between the groups.

**Methods:**

86 older adults (AD = 48; CG = 38) were evaluated using the Berg Balance Scale (BBS) and postural control was assessed by stabilometry on the Wii Balance Board ^®^ (WBB). Independent T, Mann-Whitney U-tests, Effect Size (ES) and a linear regression were performed.

**Results:**

there was a significant difference for Elliptical Area, Total Velocity, Medio-Lateral displacements with closed eyes and open eyes, antero-posterior, with closed eyes and BBS between groups. These variables showed a large effect size for BBS (-1.02), Elliptical Area (0.83) with closed eyes, Medio-Lateral (0.80, 0.96) and Total Velocity (0.92; 1.10) with eyes open and eyes closed, respectively. Regression indicated global cognition accompanied by age, gender, and diagnosis influenced postural control.

**Conclusion:**

patients with AD showed impaired postural control compared to Control Group subjects. Total Velocity with closed eyes was the most sensitive parameter for differentiating groups and should be better investigated as a possible motor biomarker of dementia in posturographic analysis with WBB.

## RESUMO.

Ferramentas clínicas adicionais devem ser investigadas para facilitar e auxiliar o diagnóstico prévio do declínio cognitivo. O controle postural piora com o envelhecimento e este fato pode estar relacionado com o comprometimento cognitivo patológico.

**Objetivo:**

comparar o equilíbrio de adultos idosos sem demência no grupo controle (GC) e com doença de Alzheimer (DA), observar as possíveis associações com as variáveis independentes (diagnóstico, idade, sexo e estado cognitivo global) e verificar as melhores análises posturográficas para determinar a diferença entre os grupos.

**Métodos:**

86 idosos (DA = 48; GC=38) foram avaliados utilizando a escala de equilíbrio Berg (EEB) e o controle postural pela estabilometria no Wii Balance Board ^®^ (WBB). Testes T independente, Mann Whitney U, o tamanho de efeito (TE) e uma regressão linear foram realizados.

**Resultados:**

houve diferença significativa para AE, VT, ML com OA e OF, AP com OF e EEB entre os grupos. Estas variáveis mostraram um TE grande para EEB (-1.02), AE (0,83) com OF, ML (0,80; 0,96) e VT (0,92; 1,10) com OA e OF, respectivamente. A regressão indicou que a cognição global acompanhada da idade, gênero e diagnóstico contribuem para as alterações do controle postural.

**Conclusão:**

pacientes com DA apresentam comprometimento do controle postural quando comparados a idosos saudáveis. A VT com OF foi o parâmetro mais sensível para diferenciar os grupos e deve ser melhor investigada como possível biomarcador motor de demência na análise posturográfica com o WBB.

The aging process is accompanied by inevitable physiological and functional decline. Genetic and environmental factors may aggravate functional impairment, especially when associated with a neurodegenerative disease.^1^ Older adults over 65 years old suffer at least one fall a year^2^ and the increase in the number of chronic-degenerative diseases in these individuals further increases this risk of falls. These events have a relationship with age and balance control, and the older the individual, the worse the stability and consequently, the greater their risk of falls.^3^

Among the neurodegenerative diseases, dementias are more prevalent, with Alzheimer’s disease (AD) the most common.^4^ Symptoms, such as impairments of memory, language, problem-solving, and other cognitive abilities, affect the ability to perform daily activities.^5^ AD is also associated with decreased mobility and balance and, hence, independence.^6^ The clinical staging of AD is classified as mild (CDR1), moderate (CDR2), or severe (CDR3) by the Clinical Dementia Rating,^7^ according to the impairment of the patient with AD.

About two-thirds of older people with cognitive impairment suffer a fall yearly,^8^ a rate three times higher than in older adults without dementia (CG).^9^ These events are usually more severe in the AD population, resulting in trauma, such as hip fractures.^10^ Elderly people with AD are more susceptible to these consequences^9^ because impairments in gait and balance,^11^ limited attention,^12^^,^ 13 use of psychotropic drugs,^14^ and behavioral changes^15^ may increase the risk of falls in this population.^16^ Static standing posture is a very important motor function for activities of daily living (ADL) and seems to be correlated with cognitive function.^17^ Therefore, it is important to detect pre-clinical manifestations of these motor impairments, such as postural control deficit, which could be a low-cost motor biomarker to aid in differential clinical diagnosis and follow-up of AD progression.

Subjective assessments of balance, which do not employ specific equipment, such as the Berg Balance Scale (BBS), are widely used. However, in spite of their ease of application and the fact they provide important information,^18^^,^ 19 they are not sensitive for detecting small changes in balance.^19^ However, stabilometry, an objective way of assessing balance, uses a force platform that is sensitive to such changes in postural control^20^ through center of pressure (CoP) tracking. In addition, there is the Wii Balance Board (WBB) (Nintendo®, Kyoto, Japan), which is similar to a force platform. A recent review showed that, despite limitations, WBB can provide valid results and has reliability characteristics similar to force platforms for static standing computerized posturography.^21^ In addition, this equipment is portable, simple to operate, and lower cost than the traditional force platform. This makes its adoption more clinically feasible, which would be useful in the clinical evaluation of patients with AD because it is a valid and reliable device.^22^^,^ 23

Although the force platform is one of the most used instruments, there is no consensus on which CoP variables should be used in the assessment of postural control. The relationship between the scores on balance tests and CoP displacement measures are moderate.^24^ Therefore, the combined use of subjective and quantitative assessments could increase the detail of the data.^19^ However, the hypothesis holds that the platform is sensitive for detecting differences in postural control between distinct groups, thereby aiding clinical decision-making. The objectives of the present study were: (1) to compare the postural control of older adults in the CG with that of AD patients using the BBS and stabilometry on the WBB platform; (2) to observe possible association with the independent variables (diagnosis, age, gender and global cognition); and (3) to verify the best posturographic analyses to determine the difference between groups.

## METHODS

### Study design and sample selection

This cross-sectional study followed the Strengthening the Reporting of Observational Studies in Epidemiology: STROBE Statement^25^ and was part of a larger study, entitled “The Effectiveness of Physical Exercise in the Treatment of Alzheimer’s Disease, Major Depression and Parkinson’s Disease” (number: 1.039.235). This study commenced in 2014 and will be run until 2019. The data collection presented in this study was performed between August 2014 and June 2017. All participants gave informed consent, which described all the research information and the contact of the researcher responsible for the study.

In this study, 86 subjects (>60 years old) were recruited (48 with AD and 38 healthy). The inclusion criteria were: subjects aged over 60 years, literate, with a previous clinical diagnosis of AD and older adults with no previous history of psychiatric disease. Exclusion criteria were: subjects with orthopedic disorders that rendered them unable to perform the necessary tests, subjects with other neurological impairments or with a diagnosis of severe stage AD (CDR3).

AD subjects were recruited at the Center for Alzheimer’s Disease and Related Disorders, Psychiatry Institute, Federal University of Rio de Janeiro (CDA-IPUB-UFRJ). The diagnosis was established according to the criteria defined for a Structured Clinical Interview for DSM Disorders (SCID), whereas the Diagnostic and Statistical Manual of Mental Disorders (DSM-V - American Psychiatric Association, 2014) was used for mental disorders.

The assessments were performed during two visits. On the first visit, all procedures were explained to the participants (CG) or their caregivers (AD). Subjects underwent anamnesis, and a global cognitive status assessment using the Mini-Mental State Examination (MMSE).^26^ On the second visit, balance tests (BBS and postural control on WBB platform) and the handgrip test were applied.

### Data sources

Postural control was evaluated by computerized posturography using stabilometry with the WBB platform. The signal was obtained at a frequency of 40 Hz and a Butterworth 8^th^ low-pass filter of 12 Hz was used to eliminate frequencies from artifacts with the Labview® software. For the analysis of postural control data, the Matlab® software (version 16) was used. The subjects positioned their feet as comfortably as possible, without exceeding shoulder width. The support base with the positioning of the feet of each subject was recorded and stored, in order to maintain the same position in the later evaluations. The evaluator instructed each subject to remain in the upright quiet posture, as still as possible, and with arms relaxed alongside the body during the evaluation. The measurements were performed six times, the first three with eyes open (EO) and the last three with eyes closed (EC). Among the three tests with EO, the test with the lowest elliptical area value of 95% of CoP^27^ was chosen (the best postural control test), and all variables were extracted from this test. The same procedure was followed for the evaluation with EC. The lowest of the three values was chosen to minimize factors which could influence balance (e. g. adaptation to quiet posture with gaze fixed on a point on the wall). Each subject was instructed to focus gaze on a fixed point for one minute (marked on the wall, at the height of the eyes and 2 m away from subject). The first 30 seconds (s) of each test were used only to adapt the assessed position. Signal uptake was performed only during the last 30 s of the test. The software stopped recording automatically, according to the measurement time. Thus, only the last 30 s of data for each test on the WBB were captured and analyzed. A rest interval of 1 minute was allowed between the tests. The variables of interest were obtained by means of the coordinates (X and Y) of the CoP. Therefore, Elliptical Area 95% (EA), Total Velocity (TotVel), antero-posterior (AP) and Medio-Lateral (ML) (both are the range of the time series of CoP), and Total Displacements (TD) with EO and EC were obtained. CoP velocity is used as the main cognitive decline-related outcome, and few studies have shown the role of other stabilometry variables in AD.^28^^,^ 29 Therefore, we included all the aforementioned stabilometry outcomes. All procedures followed the standards of validity and reliability previously published.^22^^,^ 23 These variables, as well as others commonly used in posturography, are described in Table 1, together with code examples for the Matlab programming environment. These codes assume that the CoP data in the AP and ML directions, as CPap and CPml, respectively, are variables in the Matlab environment, where corrections were also performed by regressions.

**Table 1:** Variables obtained from CoP and codes to calculate in Matlab.

Variables	Matlab codes
Anteroposterior displacements	$\mathrm{CoP}\;\mathrm{ap}=\mathrm{filtfilt}\left(b,a,\mathrm{file}\left(∶,2\right)\right)110;$
	$\mathrm{CoP}\;\mathrm{AP}=\left(\max\left(\mathrm{CoP}\;\mathrm{ap}\right)-\min\left(\mathrm{CoP}\;\mathrm{ap}\right)\right);$
Mediolateral displacements	$\mathrm{CoP}\;\mathrm{ml}=\mathrm{filtfilt}\left(b,a,\mathrm{file}\left(∶,1\right)\right)\mathrm{./}10;$
	$\mathrm{CoP}\;\mathrm{ML}=\left(\max\left(\mathrm{CoP}\;\mathrm{ml}\right)-\min\left(\mathrm{CoP}\;\mathrm{ml}\right)\right);$
Total displacements	DOT=sumsqrtCoPAP.^2+CoPML.^2;
Total velocity	TotalVel=sumsqrtdiffCoPAP.^2+diffCoPML.^2★freqlengthCoPAP;
Elliptical Area 95%	$\left[\mathrm{vec},\mathrm{vall}=\mathrm{eig}\left(\mathrm{cov}\left(\mathrm{CoP}\;\mathrm{AP},\mathrm{CoP}\;\mathrm{ML}\right)\right)\right.;$
	$\mathrm{EA}=\mathrm{pi}★\mathrm{prod}\left(2.4478★\mathrm{sqrt}\left(\mathrm{svd}\left(\mathrm{vall}\right)\right)\right);$

The evaluation of balance was performed with the BBS, which evaluates the performance of functional balance on 14 items common to basic activities of daily living. Each item has an ordinal score of five alternatives ranging from 0 to 4 points. Therefore, the maximum score is 56 points, where the higher the score, the better balance and, consequently, the lower the risk of falling.^30^

Handgrip strength test was performed with a dynamometer (TAKEI®, Japan) as a way of assessing muscle strength, in order to estimate sarcopenia. Three repetitions were performed with each hand and the subject was instructed to perform maximum possible flexion of the fingers for five seconds. The highest value for each hand was considered.^31^ In addition, total body mass (Kg) and body mass index (BMI) were measured.

### Statistical methods

The normality and homoscedasticity of the sample were analyzed by the *Kolmogorov-Smirnov* test and the *Levene test*, respectively. Data are expressed as mean ± standard deviation and median (interquartile range) for normal and non-normal data, respectively. To evaluate differences between groups, the independent T-test for parametric variables and Mann-Whitney U-test were applied for non-parametric variables. The Chi-square test was used for categorical variable (gender). Logs were used for all stabilometry variables. Effect Size (ES) was calculated for the balance variables (BBS and postural control) in order to estimate the dependent variable with the greatest effect on group differentiation.^32^ In addition, linear regression was processed for dependent variables that had large ES (≥0.8) and the enter method applied to identify which independent variables (diagnosis, age, gender and MMSE) most contributed to changes in postural control. Bonferroni adjustment was used when multiple comparisons were done. The software used for all statistical analyses was SPSS version 19 and the accepted significance level was p ≤ 0.05.

## RESULTS

The characteristics of the sample are presented in Table 2. As expected, data analysis showed that the groups differed in age (CG < AD), education (AD < CG), global cognitive status (AD < CG), and CDR, because these are the main risk factors for the disease. However, the groups were similar in relation to total body mass, height, Body Mass Index (BMI), handgrip strength, and the number of falls per year.

**Table 2:** Descriptive analyses of samples.

Variable	CG (n=38)	AD (n=48)	t/U	p-value
Age, years	73.6 ± 9.11	80.2 ± 7.61	–3.66^a^	<0.001*
Education, years	12 (6.3)	9 (7)	–3.21^b^	0.001*
Gender, %females	92.1%	56.3%	16,79^c^	<0.001*
Total body mass, kg	64.5 (13.5)	64 (13.5)	0.33^b^	0.730
Height, m	1.58 (0.1)	1.57 (0.2)	–0.14^b^	0.880
BMI, kg/m^2^	25.96 ± 2.89	25.65 ± 4.23	0.38^a^	0.705
Handgrip, kgf	23.3 (8.0)	21.5 (10.1)	–0.80^b^	0.420
Falls, nº/year	0 (0.5)	0 (1.0)	–1.73^b^	0.080
MMSE, score	29 (2.0)	20 (6.5)	–7.45^b^	<0.001 *
CDR, score	0	1 (1)	–8.31^c^	<0.001*

CG: control group; AD: Alzheimer disease; BMI: Body mass index; MMSE: Mini-Mental State Exam; CDR: Clinical Dementia Rating; mean ± standard deviation; median (interquartile range);

^a^ t-test of independent samples;

^b^ Mann-Whitney U test;

^c^ Chi-Square;

* p < 0.05.

Comparison of postural control and balance of the groups showed better performances in the CG group for all variables except TD-OE and TD-CE and CoP AP-OE, which did not differ significantly. On the ES analysis, a large effect was observed for TotVel and CoP ML variables with OE and CE, EA-CE and BBS (Table 3). The best posturographic analyses results were observed for TotVel-CE and BBS with ES (1.10 and -1.02), respectively.

**Table 3:** Comparison between groups in the log stabilometry measurements (WBB) and balance (BBS).

Dependent variables (Log)	CG (n=38)	AD (n=48)	t/U	p-value	ES (d)
BBS, Score	56 (1.0)	54 (4.0)	–4.56^a^	<0.0010* ^#^	–1.02**
EA-OE, cm^2^	0.59 ± 0.68	1.15 ± 0.83	–3.39^a^	0.001* ^#^	0.73
EA-CE, cm^2^	2.35 (1.97)	3.62 (5.85)	–3.44^b^	0.001* ^#^	0.83**
CoPTD-OE, cm	4.65 ± 0.46	4.75 ± 0.46	–0.99^a^	0.321	0.22
CoPTD-CE, cm	4.81 ± 0.45	4.93 ± 0.44	–1.15^a^	0.250	0.25
TotVel-OE, cm/s	0.73 ± 0.30	1.01 ± 0.31	–4.29^a^	<0.001* ^#^	0.92**
TotVel-CE, cm/s	0.88 ± 0.31	1.28 ± 0.40	–5.06^a^	<0.00* ^#^	1.10**
CoPAP-OE, cm	1.31 ± 0.35	1.44 ± 0.40	–1.52^a^	0.131	0.34
CoPAP-CE, cm	1.47 ± 0.38	1.68 ± 0.41	–2.51^a^	0.014* ^#^	0.53
CoPML-OE, cm	0.82 (0.41)	1.03 (0.69)	–3.41^b^	0.001* ^#^	0.80**
CoPML-CE, cm	0.92 (0.35)	1.29 (0.87)	–4.18^b^	<0.001* ^#^	0.96**

CG: control group; AD: Alzheimer disease; BBS: Berg Balance Scale; EA: elliptical area; CoP TD: Total CoP displacement; TotVel: total velocity; AP: antero-posterior; ML: mediolateral; OE: open eyes; CE: closed eyes; CoP: center of pressure; mean ± standard deviation; Median (Interquartile Range);

a t-test of independent samples;

b Mann-Whitney U test; ES (d): effect size;

* p ≤ 0.05;

** d ≥ 0.80;

# statistically significant after Bonferroni adjust.

Although the linear regression analysis (with confounding independent variables identified given in Table 2) showed a significant contribution to the model of all posturographic variables with large ES (BBS, EA-CE, TotVel-OE, TotVel-CE, CoP ML-OE, CoP ML-CE), the two largest predictions were also observed with TotVel-CE and BBS. The model with diagnosis, MMSE, gender and age together, explains about 40% of the result on the BBS, with age showing a significant and inversely proportional contribution to the result. With TotVel-CE, the same model explains 24% of the result, with the MMSE and diagnosis having the largest contribution plots. In addition, we have the model in question, with a prediction of 18% for EA-CE and a significant proportion for gender (Table 4). It is important to emphasize that some outcomes were not normally distributed (e. g. BBS), which may influence the results that exhibit a small mean significant difference.

**Table 4:** Linear regression for the stabilometry measurements (WBB) and balance (BBS) controlled for independent variables.

	BBS (R^2^ = 0.409; p < 0.001*)			EA-CE (R^2^ = 0.188; p = 0.003*)			TotVel-OE (R^2^ = 0.229; p < 0.001*)			TotVel-CE (R^2^ = 0.242; p < 0.001*)			CoPML-OE (R^2^ = 0.200; p < 0.002*)			CoPML-CE (R^2^ = 0.190; p = 0.001*)	
	β	p		β	p		β	p		β	p		β	p		β	p
Diagnoses	–0.181	0.207		0.193	0.248		0.175	0.284		0.210	0.185		0.139	0.401		0.188	0.247
MMSE	0.169	0.251		–0.083	0.620		–0.169	0.302		–.0279	0.081		–0.118	0.479		–0.159	0.330
Age	–0.455	p < 0.001*		0.009	0.937		0.169	0.122		0.052	0.618		0.077	0.486		0.081	0.455
Gender	–0.43	0.660		–0.251	0.029*		–0.122	0.268		–0.086	0.421		–0.255	0.026		–0.197	0.077

BBS: Berg Balance Scale; EA-CE: elliptical area-closed eyes; TotVel-OE: total velocity-open eyes; TotVel-CE: total velocity-close eyes; CoP ML-OE: center of pressure medio lateral-open eyes; CoP ML-CE: center of pressure medio lateral-close eyes; R2: determination coefficient; b: partial regression coefficients;

* p ≤ 0.05.

## DISCUSSION

The present study found greater impairment of postural control among patients with AD in area, velocity, and ML displacements of CoP with OE and CE. Balance measured with the clinical BBS also proved lower in AD than in CG older adults. However, age had a significant impact on the BBS measure. The TotVel-CE had the highest ES in the comparisons between groups and was not influenced by age and gender in the posturographic analysis.

In a recent review, all of these measures had previously been observed as variables capable of differentiating the studied groups, but were obtained using different balance assessment tools.^13^ Our study shows that each of these measures can be obtained using the WBB. Our findings corroborate results of Deschamps, Beauchet^29^ and Mignardot, Beauchet^28^ , who found an increase in the average absolute maximum velocity (AAMV) of individuals with cognitive impairment and patients with AD, suggesting that velocity may be the key to identifying a new biomarker for early cognitive dysfunction. This result is expected because velocity is associated with displacement and its time. Patients with AD have greater difficulty remaining static and move more until they reach stability, featuring a displacement greater than CG older adults on the same time measure, thus also showing a higher speed.

The ML displacement of the CoP followed the velocity result, despite the different effect size between variables. The only result that did not show a difference between groups was the CoP AP-OE and TD with OE and CE. AP-OE displacement of the CoP results diverge from the findings of Leandri, Cammisuli^33^ who showed a significant difference for this postural control analysis between healthy and AD patients. Our findings corroborated results of Andrade et al.^34^ who also found no differences between groups for TD.

As expected, we found worse balance in the AD group evaluated by the BBS. These findings partially reflect the results of Kato-Narita, Nitrini^35^ who found worse balance in patients with moderate AD compared to healthy older adults, but no difference between healthy older adults and mild AD patients. We suggest that postural control is impaired even before important cognitive complaints manifest, and can be investigated as a biomarker for differential diagnosis and for following up response to propaedeutic and therapeutic interventions in neurocognitive disorders among older adults.

Several circuitries and brain areas may contribute to explain the poor performance of postural control in AD. Visual circuitry influenced postural control, since we observed differences between OE and CE conditions. Postural sway tended to increase with CE in both groups, but was worse in the AD group. This is in agreement with the findings of Leandri, Cammisuli,^33^ which showed a worsening in postural control with CE for AP sway among HE, MCI, and AD groups. However, the authors pointed to the possibility of some information failure in other sensory systems besides vision, such as vestibular and somatosensory, which are also responsible for balance. In addition, sensory-motor processing with constant feedback is necessary for proper activation of the specific skeletal muscle to maintain optimal posture.^36^ Another important circuitry associated with postural control is the hippocampus-entorhinal cortex, areas that are degenerated in the early stages of AD.^37^ Thus, in addition to the expected memory impairment in AD, changes in postural control may be observed in the prodromal stages of the disease.^38^

The present study has some limitations that should be considered. The small sample, as well as the non-division of the disease stages, may have influenced the results Although we tried to adjust for differences between groups in age and gender, it is important to consider the influence of these confounding variables for posturographic analysis. Furthermore, the cross-sectional design precluded conclusions regarding the cause-and-effect relationship, and prospective cohort studies are necessary to better understand the losses of postural control in AD prodromal stages. Considering the progress of AD, dynamic posturography may be investigated primarily to differentiate MCI and we, therefore, suggest this test for future studies in the field. We understand that cognitive impairment is associated with postural control, leading to a deficiency in the sensory-motor strategy to be adopted and, thus, in the response to postural stability. Small changes in postural control, detected early, even in the absence of important cognitive complaints, may be associated with some change in cortical-subcortical circuitry. In the present study, TotVel with closed eyes, measured by the WBB, was the most sensitive parameter for differentiating older adults in the CG and patients with AD and should be further investigated as a possible motor biomarker for dementia conditions. To the best of our knowledge, this was the first study investigating postural control through these variables using the WBB platform, an ecological tool given its low cost and ease-of-application.

In conclusion, we conclude that posturographic ana lysis measures to differentiate between older adults in the CG and AD groups can be obtained using the WBB The TotVel variable seems to be more responsive and may not be influenced by age or gender in these groups.
